# Plantar heel pain in middle-aged and older adults: population prevalence, associations with health status and lifestyle factors, and frequency of healthcare use

**DOI:** 10.1186/s12891-019-2718-6

**Published:** 2019-07-20

**Authors:** Martin J. Thomas, Rebecca Whittle, Hylton B. Menz, Trishna Rathod-Mistry, Michelle Marshall, Edward Roddy

**Affiliations:** 1 Primary Care Centre Versus Arthritis, Research Institute for Primary Care and Health Sciences, Keele University, Keele, Staffordshire ST5 5BG UK; 2 Haywood Academic Rheumatology Centre, Midlands Partnership NHS Foundation Trust, Haywood Hospital, Burslem, Staffordshire ST6 7AG UK; 3 Keele Clinical Trials Unit, David Weatherall Building, Keele University, Keele, Staffordshire ST5 5BG UK; 4 Discipline of Podiatry and La Trobe Sport and Exercise Medicine Research Centre, School of Allied Health, Human Services and Sport, La Trobe University, Bundoora, Victoria 3086 Australia

**Keywords:** Plantar heel pain, Prevalence, Epidemiology

## Abstract

**Background:**

The objectives of this study were to estimate the population prevalence and distribution of plantar heel pain in mid-to-older age groups, examine associations with selected health status and lifestyle factors, and report the frequency of healthcare use.

**Methods:**

Adults aged ≥50 years registered with four general practices were mailed a health survey (*n* = 5109 responders). Plantar heel pain in the last month was defined by self-reported shading on a foot manikin, and was defined as disabling if at least one of the function items of the Manchester Foot Pain and Disability Index were also reported. Population prevalence estimates and associations between plantar heel pain and demographic characteristics, health status measures and lifestyle factors were estimated using multiple imputation and weighted logistic regression. Healthcare professional consultation was summarised as the 12-month period prevalence of foot pain-related consultation.

**Results:**

The population prevalence of plantar heel pain was 9.6% (95% CI: 8.8, 10.5) and 7.9% (7.1, 8.7) for disabling plantar heel pain. Occurrence was slightly higher in females, comparable across age-groups, and significantly higher in those with intermediate/routine and manual occupations. Plantar heel pain was associated with physical and mental impairment, more anxiety and depression, being overweight, a low previous use of high-heeled footwear, and lower levels of physical activity and participation. The 12-month period prevalence of foot pain-related consultation with a general practitioner, physiotherapist or podiatrist/chiropodist was 43.0, 15.1 and 32.8%, respectively.

**Conclusions:**

Plantar heel pain is a common, disabling symptom among adults aged 50 years and over. Observed patterns of association indicate that in addition to focused foot-specific management, primary care interventions should also target more general physical and psychological factors that could potentially act as barriers to treatment adherence and recovery.

**Electronic supplementary material:**

The online version of this article (10.1186/s12891-019-2718-6) contains supplementary material, which is available to authorized users.

## Background

Plantar heel pain is one of the most common musculoskeletal conditions affecting the lower limb, and is known to affect both physically active and sedentary individuals. In athletes, the prevalence of plantar heel pain has been estimated at between 5 and 18% [1], making it one of the most common foot-related running-related injuries [2]. Prevalence estimates from population-based studies vary depending on the age of the sample and case definition used. In Australia, the North West Adelaide Health Study of 3,206 people aged 18 years and over reported that 3.6% of the sample had plantar heel pain [3]. In the USA, the Feet First study of 784 people aged 65 years and over found that 6.9% reported tenderness in the plantar fascia and 4.2% reported tenderness in the plantar heel pad [4], while the Framingham study of 3,378 people aged 18 years and over reported the prevalence of heel pain to be 7.3% [5].

Plantar heel pain is a common reason for health professional consultation. In the USA, it has been estimated that management of plantar heel pain accounts for 1 million physician visits per year [6], with the associated annual economic burden calculated at US$284 million [7]. In the UK, 12.1% of all musculoskeletal foot and ankle consultations in primary care in 2006 were related to heel pain, with 7.5% specifying plantar fasciitis [8]. Allied health professionals are also frequently consulted by people with plantar heel pain. In Australia, plantar heel pain was the main presenting complaint in 10% of patients attending a metropolitan university podiatry clinic [9], while in the USA, 7.1% of patients with plantar heel pain are referred to a physical therapist [10].

Despite substantial personal disability and societal burden, the aetiology of plantar heel pain remains unclear [11]. In addition to foot-level factors such as pronated foot type, [12, 13] limited ankle joint dorsiflexion [13], first metatarsophalangeal joint dorsiflexion [14] and reduced muscle strength [13], studies have shown that plantar heel pain is associated with a range of person-level factors, including increased body mass index (BMI) [12, 13], depression, anxiety and stress [15], and occupations requiring prolonged periods of standing [16]. Although these findings from cross-sectional studies do not necessarily infer causation, they support the view that plantar heel pain is a multifactorial problem [17].

Whilst previous studies highlight the complexity and impact of plantar heel pain, these analyses were not conducted within representative samples and used a variety of case definitions. To the authors’ knowledge, there has been no attempt to investigate all of these person-level aspects in one representative general population sample frame. Furthermore, the few studies that have examined consultation patterns for plantar heel pain focused on specific health professions in isolation [6, 8, 10], so the relative consultation frequency is unknown.

Therefore, the objectives of this study were to (i) estimate the prevalence of plantar heel pain and disabling plantar heel pain in a representative, population-based sample, (ii) examine associations with selected aspects of health status and lifestyle factors, and (iii) report the frequency of healthcare use associated with this condition.

## Methods

### Study design

This study uses baseline data from the Clinical Assessment Study of the Foot (CASF) [18]. CASF is a population-based prospective observational cohort study of adults aged 50 years and over, who were registered at one of four general practices in North Staffordshire, UK and invited irrespective of foot-related consultation. Ethical approval was obtained from Coventry Research Ethics Committee (REC reference number: 10/H1210/5).

### Data collection

All eligible participants were mailed a Health Survey questionnaire at baseline that obtained information on aspects of general health including Short Form-12 (SF-12) [19], Hospital Anxiety and Depression Scale (HADS) [20], Short-Form International Physical Activity Questionnaire (IPAQ) [21], Keele Assessment of Participation (KAP) [22], self-reported height and weight, self-reported frequency of previous high/low heeled footwear [18], and demographic and socio-economic characteristics (age, gender, marital status, higher education, current employment status and occupation). Specific foot pain questions included: pain in and around the foot in the past 12 months; pain, aching or stiffness in the foot in the past month [5]; number of days with foot pain in the past 12 months; the Manchester Foot Pain and Disability Index (MFPDI) [23]; and general practitioner or allied health professional consultation for foot pain in the previous 12 months. The location of foot pain experienced in the past month was obtained by shading on a foot or ankle manikin, with right and left feet illustrated separately (© The University of Manchester 2000. All rights reserved) [24]. Non-responders to the Health Survey questionnaire were sent a reminder postcard after 2 weeks and a repeat Health Survey questionnaire 4 weeks after the initial mailing.

### Case definitions

Plantar heel pain was defined as any ache or pain that had lasted 1 day or longer in one or both feet during the past month, together with self-reported shading of a predefined plantar heel region on the foot and/or ankle manikin [24, 25]. Individuals were defined as having plantar heel pain if one or both heels were affected.

Disabling plantar heel pain was defined as fulfilling the definition of plantar heel pain described above and reporting foot problems on at least one of the 10 function items of the MFPDI occurring ‘on most/every day(s)’ [23, 26]. Individuals were defined as having disabling plantar heel pain if one or both heels were affected. Disabling plantar heel pain is therefore a subgroup of the plantar heel pain definition above.

### Statistical analysis

#### Estimating the population prevalence

The population prevalence of plantar heel pain and disabling plantar heel pain were estimated using the baseline Health Survey questionnaire data. Missing item-level data for the baseline variables, arising from non-completion of individual items in the Health Survey questionnaire, were imputed using multiple imputation for all baseline responders. Estimates were weighted to account for any initial selective non-response from the eligible baseline population to the Health Survey questionnaire. The assumption that data were missing at random was accepted as reasonable.

The weighted logistic regression model included age, gender and general practice, which was available for all individuals, regardless of whether they responded to the questionnaire or not. This was used to determine a weight to reflect the likelihood that a person with a specific combination of age, gender and practice location would return the Health Survey questionnaire.

To enable comparison, the imputation model included the same auxiliary variables as we have used previously to estimate the population prevalence of symptomatic radiographic foot osteoarthritis [27, 28], with the removal of foot osteoarthritis and pain regions and the addition of the plantar heel pain regions, SF-12 [19], IPAQ [21], the KAP [22] statement “During the past 4 weeks, I have moved around outside my home, as and when I have wanted”, self-reported frequency of previous high/low heeled footwear, BMI and general practitioner or allied health professional (physiotherapist or podiatrist/chiropodist) consultation for foot pain in the previous 12 months. The original variables included were: age, gender, general practice, social class, marital status, number of days in the past 12 months with foot pain, Rasch-transformed MFPDI pain and function scores [29], individual MFPDI function items to determine disabling symptoms [26], report of pain, aching, or stiffness in the foot in the last month, SF-12 score and HADS score. The imputation model generated 15 imputed datasets and analysis was performed within each imputed dataset. The estimates from each imputed dataset were then combined using Rubin’s rules to obtain overall estimates [30].

The *mim:proportion* command was used to determine the population prevalence estimates (and 95% confidence intervals [CI]) of plantar heel pain and disabling plantar heel pain from the imputed dataset. The analyses were weighted to take into account non-response. The population prevalence was then stratified by gender, age-group and socio-economic class.

#### Associations between demographics, selected aspects of health status and lifestyle factors

Using the imputed data, logistic regression, weighted to account for non-response, was used to estimate the associations between plantar heel pain and the following variables: gender, age, socio-economic classification, SF-12 physical and mental health component scores, dichotomised at the median values into low and high physical/mental health (low physical health ≤42.6, low mental health ≤52.6), HADS anxiety and depression, categorised into normal (0–7), mild (8–10), moderate (11–14) and severe (15–21) [31], BMI calculated from self-reported height and weight (reference category < 25 kg/m^2^), self-reported lifetime recall of frequent high-heeled footwear use among females [18] (high frequency defined as reported use of high-heeled shoes on most days for at least one 10-year period between 20 and 49 years of age), short-form IPAQ physical activity levels categorised as low, moderate or high [21] and response to the KAP statement “During the past 4 weeks, I have moved around outside my home, as and when I have wanted”, with response options dichotomised as all of the time/most of the time versus some of the time/a little of the time/none of the time [22]. Crude odds ratios (ORs) (and 95% CIs) and adjusted ORs were estimated, adjusting BMI for age and gender, frequent use of high-heeled footwear for age and BMI and all other variables for age, gender and BMI.

#### Frequency of consultation for foot pain

The 12-month period prevalence of foot pain-related consultation with a general practitioner or allied health professional (physiotherapist or podiatrist/chiropodist) among adults with plantar heel pain was estimated using the imputed data. Prevalence estimates for allied health professionals were further stratified by consultation type (National Health Service [NHS] or private practice).

All analyses were conducted using STATA V.14.2 (Stata Corporation, TX, USA).

## Results

A full description of participant recruitment and characteristics in the CASF study has been reported previously [27]. Briefly, in 2010/2011, 9,344 adults aged ≥50 years were mailed a baseline Health Survey Questionnaire. Of the 9,194 eligible baseline-mailed population, 5,109 Health Surveys were returned (adjusted response 56%) [27]. Data on plantar heel pain were missing for 2.7% of participants. Missing data for the other variables of interest ranged from 1.4 to 15.2%. A supplementary table describing participant characteristics in the whole cohort and stratified by presence of plantar heel pain on non-imputed data is provided in Additional file 1.

### Population prevalence

Following multiple imputation and weighted logistic regression to account for missing data and non-response, respectively, the population prevalence of plantar heel pain in adults aged 50 years and over was 9.6% (95% CI 8.8, 10.5) and the population prevalence of disabling plantar heel pain was 7.9% (95% CI 7.1, 8.7). Therefore, the majority of those with plantar heel pain had disabling pain. Plantar heel pain was only slightly more prevalent in females and comparable across age groups, but occurrence was most common in those with routine and manual occupations (Table 1). For disabling plantar heel pain, prevalence was slightly higher in females, slightly higher in older age groups and also most common in those with routine and manual occupations.

**Table 1:** Population prevalence of plantar heel pain and disabling plantar heel pain by demographic characteristics

	Plantar heel pain	Disabling plantar heel pain
All adults aged 50+	9.6 (8.8, 10.5)	7.9 (7.1, 8.7)
Gender		
Male	9.2 (8.1, 10.4)	7.5 (6.4, 8.6)
Female	10.0 (8.8, 11.2)	8.3 (7.2, 9.4)
Age (years)		
50–64	9.8 (8.6, 11.1)	7.5 (6.4, 9.3)
65–74	9.3 (7.8, 10.9)	7.9 (6.5, 9.3)
75+	9.4 (7.5, 11.4)	8.9 (7.0, 10.8)
Age, Males		
50–64	9.7 (8.0, 11.4)	7.2 (5.7, 8.7)
65–74	8.7 (6.7, 10.8)	7.7 (5.7, 9.7)
75+	8.5 (5.7, 11.4)	8.1 (5.2, 10.9)
Age, Females		
50–64	10.0 (8.3, 11.7)	7.8 (6.3, 9.4)
65–74	9.9 (7.7, 12.2)	8.1 (6.1, 10.2)
75+	10.1 (7.5, 12.7)	9.5 (7.0, 12.0)
Socio-economic classification		
Managerial and professional	4.6 (3.3, 5.9)	3.0 (1.9, 4.1)
Intermediate occupations	8.4 (6.6, 10.3)	6.8 (5.2, 8.5)
Routine and manual	10.9 (9.7, 12.1)	9.0 (7.8, 10.1)

Estimates are percentages with 95% confidence intervals based on imputed and weighted analyses

### Associations between demographics, selected aspects of health status and lifestyle factors

After adjustment for potential confounders, positive associations were observed between plantar heel pain and lower socio-economic classification, and impaired physical and mental health measured using the SF-12. For anxiety and depression using the HADS scores, and BMI, dose-response relationships were observed, with positive associations with plantar heel pain becoming stronger with increased severity and increased BMI, respectively. Plantar heel pain was also positively associated with low physical activity measured using the Short-Form IPAQ and limitations in undertaking activity outside the home measured using the KAP (Table 2). Among females infrequent/low use of high-heeled footwear was associated with plantar heel pain. There were no associations between plantar heel pain and gender or age.

**Table 2:** Associations between plantar heel pain and selected demographics, health status and lifestyle factors

	Crude OR (95% CI)	Adjusted OR (95% CI)
Gender		
Male	1	1
Female	1.09 (0.90, 1.32)	1.05 (0.86, 1.27)^a^
Age (years)		
50–64	1	1
65–74	0.94 (0.75, 1.18)	0.99 (0.78, 1.24)^b^
75+	0.95 (0.73, 1.24)	1.12 (0.86, 1.46)^b^
Age, Males		
50–64	1	1
65–74	0.89 (0.64, 1.23)	0.94 (0.68, 1.31)^b^
75+	0.87 (0.57, 1.31)	1.01 (0.67, 1.54)^b^
Age, Females		
50–64	1	1
65–74	1.00 (0.73, 1.36)	1.03 (0.75, 1.41)^b^
75+	1.01 (0.72, 1.42)	1.21 (0.86, 1.71)^b^
Socio-economic classification		
Managerial and professional	1	1
Intermediate occupations	1.90 (1.29, 2.80)	1.87 (1.27, 2.77)^c^
Routine and manual	2.52 (1.82, 3.50)	2.51 (1.80, 3.48)^c^
SF-12 Physical Component Score		
High physical health (> 42.6)	1	1
Low physical health (≤42.6)	4.16 (3.28, 5.27)	4.01 (3.13, 5.14)^c^
SF-12 Mental Component Score		
High mental health (> 52.6)	1	1
Low mental health (≤52.6)	2.76 (2.21, 3.43)	2.58 (2.07, 3.22)^c^
HADS Anxiety		
Normal (0–7)	1	1
Mild (8–10)	2.40 (1.87, 3.07)	2.35 (1.83, 3.01)^c^
Moderate (11–14)	3.11 (2.40, 4.03)	2.95 (2.26, 3.84)^c^
Severe (15–21)	4.12 (2.97, 5.71)	3.86 (2.74, 5.42)^c^
HADS Depression		
Normal (0–7)	1	1
Mild (8–10)	2.39 (1.86, 3.07)	2.16 (1.67, 2.81)^c^
Moderate (11–14)	3.57 (2.72, 4.69)	3.08 (2.33, 4.08)^c^
Severe (15–21)	4.33 (2.84, 6.62)	3.80 (2.41, 5.98)^c^
Body mass index (kg/m^2^)		
< 25	1	1
25–29.9	1.47 (1.13, 1.91)	1.48 (1.14, 1.93)^d^
30–34.9	1.84 (1.36, 2.48)	1.85 (1.37, 2.50)^d^
≥ 35	4.44 (3.21, 6.13)	4.47 (3.23, 6.18)^d^
Self-reported frequent use of high-heeled footwear^e^		
Low	1	1
High	0.72 (0.55, 0.95)	0.73 (0.55, 0.96)^a^
Physical activity (Short-Form IPAQ)		
Low	1	1
Moderate	0.57 (0.45, 0.72)	0.61 (0.48, 0.78)^c^
High	0.46 (0.35, 0.60)	0.50 (0.38, 0.66)^c^
Keele Assessment of Participation^f^		
All of the time/most of the time	1	1
Some of the time/a little of the time/none of the time	3.10 (2.55, 3.78)	2.96 (2.40, 3.66)^c^

Based on imputed and weighted analyses

*OR* Odds ratio, *CI* Confidence Interval, *SF-12* Short Form-12, *HADS* Hospital Anxiety and Depression Scale, *IPAQ* International Physical Activity Questionnaire

^a^Estimate adjusted for age and body mass index

^b^Estimate adjusted for gender and body mass index

^c^Estimate adjusted for age, gender and body mass index

^d^Estimate adjusted for age and gender

^e^Estimate restricted to females and the exposure was defined as previous footwear (low- versus high-heeled shoes) worn on most days for at least one 10-year period between 20 and 49 years old

^f^Response to statement: During the past 4 weeks, I have moved around outside my home, as and when I have wanted

### Frequency of consultation for foot pain

Nearly two-thirds of participants with plantar heel pain had consulted a healthcare professional in the last 12 months for foot pain, with a similar number consulting a general practitioner or allied health professional. Nearly one-third of participants had consulted an NHS podiatrist/chiropodist, which was more than double the proportion who had consulted an NHS physiotherapist for foot pain. A minority of participants had consulted a health professional in private practice, and the majority of these were with a podiatrist/chiropodist (Table 3).

**Table 3:** Frequency of selected healthcare professional consultation for foot pain, among adults with plantar heel pain

Healthcare professional consulted	12-month period prevalence % (95% CI)
General practitioner	43.0 (38.4, 47.5)
Physiotherapist/podiatrist/chiropodist	41.0 (36.5, 45.6)
Any of the above	61.5 (57.1, 66.0)
Physiotherapist	
NHS	12.7 (9.5, 15.8)
Private	3.4 (1.4, 5.4)
Any of the above	15.1 (11.7, 18.4)
Podiatrist/chiropodist	
NHS	27.1 (23.0, 31.1)
Private	9.1 (6.3, 11.8)
Any of the above	32.8 (28.5, 37.1)

Based on imputed and weighted analyses

There were no large differences between imputed and non-imputed data (data not shown).

## Discussion

The aims of this study were to describe the occurrence of plantar heel pain, and examine associated health status and frequency of healthcare consultation using a representative population sample of adults from mid-older age. Our findings suggest that plantar heel pain affects approximately one in 10 adults aged 50 years and over in the general population, with approximately 80% experiencing some form of disability due to their heel pain. A point prevalence estimate of 9.6% (95% CI 8.8, 10.5) is higher than previous studies, which, despite using slightly different case definitions and age ranges, have reported estimates between 4 and 7% [3–5]. The observed minimal gender difference is also consistent with previous estimates [3–5]. Our age-stratified prevalence estimates for plantar heel pain are largely comparable with systematic review findings that chronic plantar heel pain appears to occur most commonly between the age of 40 to 59 years [32], however the prevalence of disabling plantar heel pain was slightly higher in adults aged 75 years or older in our sample. The observed positive associations with high BMI, impaired physical and mental health, more anxiety and depression, and low physical activity and participation are consistent with previous observations [12, 13, 15, 16], but extend these findings by comparing all of these person-level aspects within a single population sample frame. Taken together, these findings suggest that general physical and psychological factors could potentially act as barriers to treatment adherence and recovery in people with plantar heel pain. The observed association with low previous use of high-heeled footwear among females did not have a notable impact on prevalence when compared to males. The observed association that high-heel footwear may have a protective effect is noteworthy. One possible explanation is that slight elevation of the heel provided by certain types of footwear may serve to offload the plantar fascia at its origin on the calcaneus [33]. This arguably places less cumulative load around the plantar heel area, but may lead to foot pain and problems elsewhere in the foot and ankle. Although we defined high-heel footwear use according to report of shoewear patterns before the age of 50 years, we do not know the lifetime duration of plantar heel pain and we cannot be certain that high-heel footwear use preceded plantar heel pain onset, and that high-heeled footwear use was not modified in response to pain. The self-reported nature of this variable could also have diluted the precision of the estimate due to recall bias. Further investigation in other cohorts is warranted to explore this further.

A high proportion of participants with plantar heel pain consulted a health professional in the last 12 months for foot pain (61.5%, 95% CI 57.1, 66.0). Consultations were to general practitioners and allied health professionals in approximately equal proportions (43.0%, 95% CI 38.4, 47.5 and 41.0%, 95% CI 36.5, 45.6 respectively), but within allied health professions, consultations were more frequent for podiatrists/chiropodists (32.8%, 95% CI, 28.5, 37.1) than physiotherapists (15.1%, 95% CI, 11.7, 18.4). For participants consulting an NHS physiotherapist or podiatrist/chiropodist in the last 12 months, it is assumed that these most likely represent direct referrals from general practitioners, who are currently the principal gate-keepers to allied health professional treatment in England. Consultation studies from the USA have estimated that approximately 1 million people each year consult a physician for plantar heel pain [6], with 7.1% being referred to physical therapy [10]. More locally, a previous primary care record-based study conducted in North Staffordshire estimated that of all foot and ankle consultations during a 12-month period, 7.5% were for ‘plantar fasciitis’ and 4.6% were for ‘heel pain’ [8]. However, the authors acknowledged that this is likely to be an under-estimate, as many specific conditions were likely to be subsumed by the common use of generic Read code terms such as ‘foot pain’ and ‘ankle pain’ [8].

The main strength of this study is the large community-based population sample frame and the use of multiple imputation and weighted logistic regression to handle missing data and non-response for deriving prevalence estimates. Despite this, some caution should be applied to interpretation due to non-response. Adopting a survey-based approach has enabled the assembly or a large sample; however there are also some notable limitations related to this approach. Firstly, although plantar heel pain was defined from shading the appropriate region of a foot manikin, the majority of responders shaded more than 1 foot region. This may reflect the age of the population in this study (≥50 years), in whom multisite foot pain would not be unexpected. This could potentially explain our higher point prevalence estimate compared with previous studies examining wider age ranges [3, 5], as could our definition, which might include people with milder non-specific pain. Also observed associations are likely to be relative to pain elsewhere in the foot, under-estimating the true effect. Secondly, the local population sample frame used for this analysis is one of high socio-economic deprivation with more prevalent obesity and less ethnic diversity than the general UK population. Thirdly, the reported associations are also cross-sectional, and therefore causality cannot be inferred from these estimates. Furthermore, we have not explored the association of plantar heel pain with any specific disease groups. Fourthly, with the exception of gender and age, all variables included in the analysis were based on self-report data. We were therefore unable to assess the contribution of risk factors requiring clinical assessment, such as foot posture and range of motion. Whilst the previous footwear question has been utilised in our earlier studies [28, 34], this has not been formally validated. Although previous footwear may be susceptible to recall bias, these questions were asked separately and prior to those relating to foot pain. The underlying pathology driving the symptoms of plantar heel pain also remain unknown within this sample. Whilst the most likely cause is plantar fasciitis [8], a list of common potential differential diagnoses of plantar heel pain are presented in Table 4. Fifthly, the cardinal feature of pain on weight-bearing from rest was not a question asked within our questionnaire, and could have improved our case definition. The sensitivity of prevalence studies to case definition, in general, means that working toward consensus foot pain-related definitions would enhance future comparability and potential for pooling estimates. Finally, for the evaluation of disabling foot pain and health professional consultation, the questions pertained to general foot-related disability and consultation. Consequently, it is unknown whether the foot pain disability and consultation related specifically to the plantar heel.

**Table 4:** Differential diagnosis of plantar heel pain^a^

Diagnosis	
Plantar fasciitis	
Plantar fascia rupture	
Enthesopathies	
Calcaneal stress fracture	
Bone bruise	
Infection	
Cancer	
Paget’s disease	
Fat pad atrophy	
Blisters	
Bursitis	
Nerve syndromes (entrapment/compression)	
S1 radiculopathy	
Neuropathic pain	

^a^List adapted from [17]

## Conclusion

Plantar heel pain is a common, disabling symptom among adults aged 50 years and over. In addition to focused, foot-specific management of the condition, our observed patterns of association with health status and lifestyle factors indicate that primary care interventions should also consider general physical and psychological factors, that could potentially act as barriers to treatment adherence and recovery, and potentially facilitate prevention. Future prospective studies of plantar heel pain are warranted to confirm if the associations identified could be causal.

## Additional file

Additional file 1: Descriptive characteristics of cohort and stratified by presence of plantar heel pain, using complete case data. (DOCX 20 kb)

## Data Availability

The datasets used and/or analysed during the current study are available from the corresponding author on reasonable request.

## Abbreviations

BMI: Body Mass Index
CASF: Clinical Assessment Study of the Foot
CI: Confidence Intervals
HADS: Hospital Anxiety and Depression Scale
IPAQ: International Physical Activity Questionnaire
KAP: Keele Assessment of Participation
MFPDI: Manchester Foot Pain and Disability Index
NHS: National Health Service
OR: Odds Ratio
SF-12: Short-Form 12
UK: United Kingdom
USA: United States of America
