# Supplementary material for: Plantar heel pain in middle-aged and older adults: population prevalence, associations with health status and lifestyle factors, and frequency of healthcare use
Source: BMC Musculoskelet Disord. 2019 Jul 20;20:337. doi: 10.1186/s12891-019-2718-6 (PMC6642587; doi:10.1186/s12891-019-2718-6)
Supplement: Supplementary file 1 — Descriptive characteristics of cohort and stratified by presence of plantar heel pain, using complete case data. (DOCX 20 kb) [file 12891_2019_2718_MOESM1_ESM.docx]

**Additional File 1. Descriptive characteristics of cohort and stratified by presence of plantar heel pain,**

**using complete case data.**

|  | Whole cohort  N=5109  N (%) | No plantar heel pain  N=4481  N (%) | Plantar heel pain  N=449  N (%) |
| --- | --- | --- | --- |
| Gender |  |  |  |
| Male | 2439 (47.74) | 2160 (48.20) | 209 (46.55) |
| Female | 2670 (52.26) | 2321 (51.80) | 240 (53.45) |
| Age (years) |  |  |  |
| 50-64 | 2563 (50.17) | 2275 (50.77) | 237 (52.78) |
| 65-74 | 1530 (29.95) | 1349 (30.10) | 133 (29.62) |
| 75+ | 1016 (19.89) | 857 (19.13) | 79 (17.59) |
| Age, Males |  |  |  |
| 50-64 | 1263 (51.78) | 1126 (52.13) | 117 (55.98) |
| 65-74 | 756 (31.00) | 670 (31.02) | 62 (29.67) |
| 75+ | 420 (17.22) | 364 (16.85) | 30 (14.35) |
| Age, Females |  |  |  |
| 50-64 | 1300 (48.69) | 1149 (49.50) | 120 (50.00) |
| 65-74 | 774 (28.99) | 679 (29.25) | 71 (29.58) |
| 75+ | 596 (22.32) | 493 (21.24) | 49 (20.42) |
| Socio-economic classification |  |  |  |
| Managerial and professional | 1011 (22.14) | 947 (23.36) | 45 (11.72) |
| Intermediate occupations | 887 (19.43) | 793 (19.56) | 71 (18.49) |
| Routine and manual | 2668 (58.43) | 2314 (57.08) | 268 (69.79) |
| SF-12 Physical Component Score |  |  |  |
| High physical health (>42.6) | 2340 (52.22) | 2205 (55.37) | 87 (22.96) |
| Low physical health (≤42.6) | 2141 (47.78) | 1777 (44.63) | 292 (77.04) |
| SF-12 Mental Component Score |  |  |  |
| High mental health (>52.6) | 2309 (51.53) | 2141 (53.77) | 113 (29.82) |
| Low mental health (≤52.6) | 2172 (48.47) | 1841 (46.23) | 266 (70.18) |
| HADS Anxiety |  |  |  |
| Normal (0-7) | 3037 (60.66) | 2782 (63.08) | 160 (36.45) |
| Mild (8-10) | 968 (19.33) | 823 (18.66) | 117 (26.65) |
| Moderate (11-14) | 697 (13.92) | 566 (12.83) | 104 (23.69) |
| Severe (15-21) | 305 (6.09) | 239 (5.42) | 58 (13.21) |
| HADS Depression |  |  |  |
| Normal (0-7) | 3637 (72.59) | 3316 (75.16) | 219 (49.89) |
| Mild (8-10) | 760 15.17) | 634 (14.37) | 104 (23.69) |
| Moderate (11-14) | 470 (9.38) | 357 (8.09) | 85 (19.36) |
| Severe (15-21) | 143 (2.85) | 105 (2.38) | 31 (7.06) |
| Body mass index (kg/m^2^) |  |  |  |
| <25 | 1684 (34.64) | 1528 (35.78) | 97 (22.25) |
| 25-29.9 | 1948 (40.07) | 1729 (40.49) | 167 (38.30) |
| 30-34.9 | 840 (17.28) | 724 (16.96) | 85 (19.50) |
| ≥35 | 389 (8.00) | 289 (6.77) | 87 (19.95) |
| Self-reported frequent use of high-heeled footwear^a^ |  |  |  |
| Low | 887 (40.30) | 770 (39.49) | 98 (48.51) |
| High | 1314 (59.70) | 1180 (60.51) | 104 (51.49) |
| Physical activity (Short-Form IPAQ) |  |  |  |
| Low | 1119 (25.83) | 929 (24.14) | 144 (37.99) |
| Moderate | 1820 (42.01) | 1638 (42.56) | 145 (38.26) |
| High | 1393 (32.16) | 1282 (33.31) | 90 (23.75) |
| Keele Assessment of Participation^b^ |  |  |  |
| All of the time/most of the time | 3839 (76.22) | 3494 (78.87) | 242 (54.50) |
| Some of the time/a little of the time/none of the time | 1198 (23.78) | 936 (21.13) | 202 (45.50) |

Based on complete case analysis

SF-12, Short Form-12; HADS, Hospital Anxiety and Depression Scale; IPAQ, International Physical Activity Questionnaire; N, number of participants

^a^Question restricted to females and the exposure was defined as previous footwear (low- versus high-heeled shoes) worn on most days for at least one 10-year period between 20 and 49 years old

^b^Response to statement: During the past 4 weeks, I have moved around outside my home, as and when I have wanted
